# The Impact of Prostate Cancer Upgrading and Upstaging on Biochemical Recurrence and Cancer-Specific Survival

**DOI:** 10.3390/medicina56020061

**Published:** 2020-02-04

**Authors:** Arnas Bakavičius, Mingailė Drevinskaitė, Kristina Daniūnaitė, Marija Barisienė, Sonata Jarmalaitė, Feliksas Jankevičius

**Affiliations:** 1 Institute of Clinical Medicine, Faculty of Medicine, Vilnius University, 03101 Vilnius, Lithuania; m.barisiene@gmail.com (M.B.); feliksas.jankevicius@santa.lt (F.J.); 2 Vilnius University Hospital Santaros Kinikos, 08661 Vilnius, Lithuania; 3 National Cancer Institute, 08660 Vilnius, Lithuania; vailaomalyn@yahoo.com (K.D.); sonata.jarmalaite@nvi.lt (S.J.); 4 Faculty of Medicine, Vilnius University, 03101 Vilnius, Lithuania; ming.drevinskaite@gmail.com; 5 Institute of Biosciences, Life Sciences Center, Vilnius University, 10257 Vilnius, Lithuania

**Keywords:** prostate cancer, upgrading, upstaging, biochemical recurrence, metastases, survival

## Abstract

*Background and Objectives:* Significant numbers of prostate cancer (PCa) patients experience tumour upgrading and upstaging between prostate biopsy and radical prostatectomy (RP) specimens. The aim of our study was to investigate the role of grade and stage increase on surgical and oncological outcomes. *Materials and Methods:* Upgrading and upstaging rates were analysed in 676 treatment-naïve PCa patients who underwent RP with subsequent follow-up. Positive surgical margin (PSM), biochemical recurrence (BCR), metastasis-free survival (MFS), overall (OS) and cancer specific survival (CSS) were analysed according to upgrading and upstaging. *Results:* Upgrading was observed in 29% and upstaging in 22% of PCa patients. Patients undergoing upgrading or upstaging were 1.5 times more likely to have a PSM on RP pathology. Both upgrading and upstaging were associated with increased risk for BCR: 1.8 and 2.1 times, respectively. Mean time to BCR after RP was 2.1 years in upgraded cases and 2.7 years in patients with no upgrading (*p* < 0.001), while mean time to BCR was 1.9 years in upstaged and 2.8 years in non-upstaged cases (*p* < 0.001). Grade and stage increase after RP were associated with inferior MFS rates and ten-year CSS: 89% vs. 98% for upgrading (*p* = 0.039) and 87% vs. 98% for upstaging (*p* = 0.008). *Conclusions:* Currently used risk stratification models are associated with substantial misdiagnosis. Pathological upgrading and upstaging have been associated with inferior surgical results, substantial higher risk of BCR and inferior rates of important oncological outcomes, which should be considered when counselling PCa patients at the time of diagnosis or after definitive therapy.

## 1. Introduction

Globally, prostate cancer (PCa) is the second most common cancer in men, with 1.3 million incident cases and 416 000 deaths per year [1]. PCa is a heterogeneous disease in various aspects, including morphological manifestation, clinical course, and molecular features [2]. Usually, approximately three separate tumours arise in the gland [3], and several grading systems have been proposed for morphological evaluation of the disease [4]. Up to date, more than 100 molecular biomarkers have been identified in PCa [5], showing molecular complexity of PCa. As a reflection of this heterogeneity, clinical manifestation of PCa varies from indolent localised to aggressive metastatic disease.

PCa characterisation is still based on the needle biopsy, where the Gleason grading system with clinical tumour staging provide the strongest prognostic power for oncological outcomes after treatment with curative intent. Knowing such heterogeneity of the disease and diagnostic limitations of prostate biopsy, it is not surprising that a significant number of PCa patients undergoing radical prostatectomy are upgraded and upstaged [6].

The objective of the present study was to characterise the rates of pathological upgrading and upstaging after radical prostatectomy (RP) and investigate their role on surgical as well as oncological outcomes, including positive surgical margin (PSM), biochemical recurrence (BCR), metastases-free survival (MFS), overall and cancer-specific survival (OS and CSS, respectively).

## 2. Materials and Methods

This study is a part of a large-scale PCa biomarker research that started in 2008, performed after approval by the Lithuanian Bioethics Committee (2007-11-23 No. 50 and 2011-09-07 No. 6B-11-275). In the present study treatment-naïve patients with histologically confirmed localised PCa (≥ 10-core systematic biopsies by transrectal approach) who underwent open RP at Vilnius University Hospital Santaros Klinikos between January 2008 and December 2014 were included. This subgroup is a part of a large cohort involved in the biomarker-based upgrading and upstaging study [7], with available post-operative follow-up. The metastatic disease of intermediate and high-risk PCa was excluded by bone-scan and computer tomography preoperatively. All patients were followed-up subsequently at the outpatient clinic of the same institution. Metastatic disease in postoperative setting was evaluated by bone-scan and computer tomography or magnetic resonance imaging. All data regarding follow-up were collected retrospectively from postoperative medical records up to September 2019. The data regarding survival were obtained from the State Register of Death Cases and Their Causes, by the Institute of Hygiene under the Ministry of Health of the Republic of Lithuania (2019-10-21 No. (9.20) 01-517). Previous androgen-deprivation therapy, active-surveillance and history of urothelial carcinoma were considered as exclusion criteria.

Gleason score was evaluated according to the 2005 Guidelines of International Society of Urological Pathology (ISUP) and ISUP grade groups were assigned according to ISUP 2014 recommendations [8,9]. As defined previously [7], upgrading was considered when any increase of ISUP grade group between prostate biopsy (cISUP) and RP pathology (pISUP) was detected, whereas upstaging was confirmed if a patient was pathologically diagnosed with advanced disease (≥pT3) when clinically unsuspected. PSM was defined as the presence of tumour cells at the inked margin on the inspection under microscopy [10]. BCR following RP was defined as a postoperative prostate-specific antigen (PSA) >0.2 ng/mL with a subsequent confirmatory value [11]. MFS was defined as the time from RP to confirmed evidence of distant metastases on imaging. PCa overall survival (OS) after RP was defined as a time from RP to death from any cause. PCa specific survival (CSS) after RP was defined as a time from RP to death at the time of progressive metastatic disease. Patients who had died without BCR or with BCR and PSA <1.0 ng/mL with metastatic-free disease were classified as dying from other causes.

Statistical analysis as well as reporting and interpretation of the results were conducted according to the established guidelines [12]. Continuous variables are expressed as means with standard deviation (SD). Data for categorical variables are presented as frequencies and percentages. Continuous variables were checked for normal distribution by Shapiro–Wilk statistics and compared them by the *t* test when normally distributed or the Mann–Whitney U test for non-normally distributed variables. Pearson’s χ^2^ and Fisher exact tests were used for comparison of categorical variables, as appropriate. Odds ratios (OR) with 95% confidence intervals (CI) were calculated using logistic regression analysis. Kaplan–Meier curves were depicted and Log Rank (Mantel–Cox) test was applied to support the survival analyses. All statistical tests were performed using SPSS software (IBM Corp., Armonk, NY, USA). *P*-value of <0.050 was considered significant.

## 3. Results

### 3.1. Study Population

Overall, 676 patients were included into the study. Clinico-pathological characteristics of the study cohort are summarized in Table 1.

### 3.2. Upgrading, Upstaging and Surgical Margin

Upgrading was observed in 29.1% (197/676) and upstaging in 22.0% (149/676) of PCa patients undergoing RP. The total misclassification rate, when at least either upgrading or upstaging was detected, was 41.7% (282/676). Among the upgraded cases 85.3% (168/197), 10.2% (20/197), 2.0% (4/197) and 2.5% (5/197) of PCa patients were initially diagnosed with cISUP 1, 2, 3 and 4 disease, respectively. The majority of patients (73.6%, 145/197) were upgraded to pISUP grade group 2. Patients initially diagnosed with cT1c cancer dominated among the upstaged cases (47.0%; 70/149).

Positive surgical margin was detected in 32.1% (217/676) of PCa patients undergoing RP. According to prostate anatomy, apex was the most common site for PSM – 56.0% (108/193), followed by postero-lateral position—48.2% (93/193), base—15.0% (29/193) and seminal vesicles—4.7% (9/193).

The patients whose cancer was upgraded post RP more commonly had PSM (41.6%, 82/197) as compared to patients with no upgrading (28.2%, 135/479; *p* = 0.001). Upstaging after RP was also associated with PSM, where 44.3% (66/149) of PCa patients with upstaging and 28.7% (151/527) with no upstaging had been reported with PSM (*p* <0.001).

### 3.3. Biochemical Recurrence

BCR-only was diagnosed to 25.7% (174/676) of PCa patients after RP. At the time of BCR detection 77.3% (126/163) of the patients presented with PSA value <0.5 ng/mL, 12.3% (20/163) – with PSA 0.5–2.0 ng/mL and 10.4% (17/163) – with PSA >2.0 ng/mL. The mean follow-up time of patients without BCR was 46.8 months (SD: 36.3).

**Figure 1:**
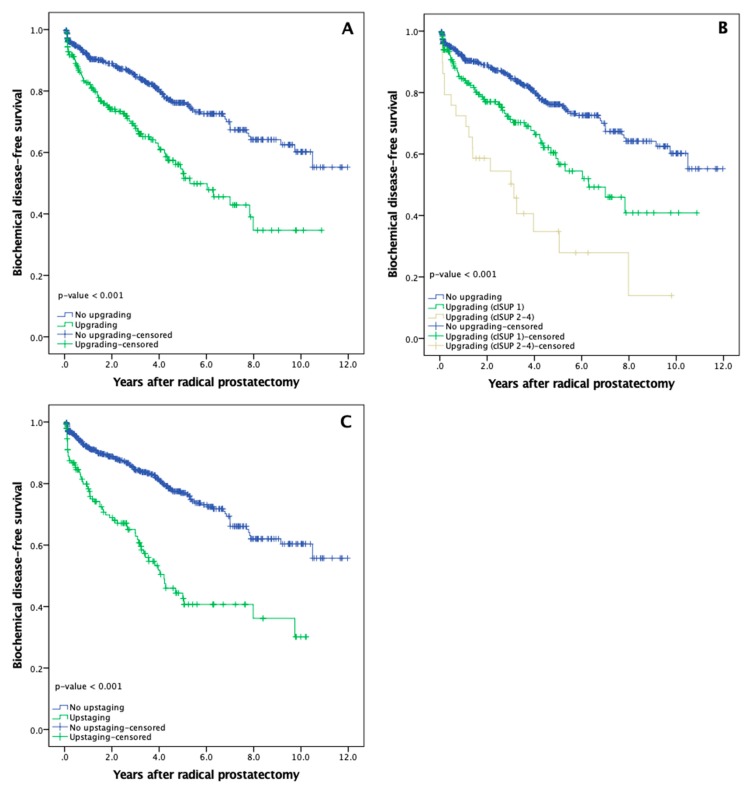
Prostate cancer biochemical disease-free survival rates after radical prostatectomy: (**A**) Biochemical disease-free survival according to upgrading (all clinical International Society of Urological Pathology (cISUP) grade groups); (**B**) Biochemical disease-free survival for patients with no upgrading (blue line), upgrading from cISUP grade group 1 (green line) and upgrading from cISUP grade group 2–4 (yellow line); (**C**) Biochemical disease-free survival according to upstaging.

BCR was diagnosed to 37.6% (74/197) of PCa patients whose cancer was upgraded post RP, while only to 20.9% (100/479) of patients with no upgrading (*p* <0.001). Mean time to BCR after RP was 2.1 years (SD: 2.0) in upgraded cases and 2.7 years (SD: 2.5) in patients with no upgrading (Figure 1A; *p* <0.001). Patients who were upgraded from clinically low risk (cISUP 1) disease showed more favourable BCR rates as compared to patients with clinically diagnosed intermediate or high risk (cISUP 2–4) PCa (Figure 1B; *p* <0.001).

Upstaging after RP was also associated with BCR, where 43.6% (65/149) of PCa patients undergoing upstaging in contrast to 20.7% (109/527) of patients without upstaging were diagnosed with BCR (*p* <0.001). Mean time to BCR after RP was 1.9 years (SD: 2.0) in upstaged and 2.8 years (SD: 2.5) in non-upstaged cases (Figure 1C; *p* <0.001).

In logistic regression analysis PSM showed the highest OR for BCR (2.29 (1.55–3.40), *p* < 0.001). According to this model, the ORs for upgrading and upstaging were 1.92 (1.29–2.86) and 2.14 (1.39–3.27), respectively (Table 2; all *p* < 0.001).

### 3.4. Metastasis-Free Survival

**Figure 2:**
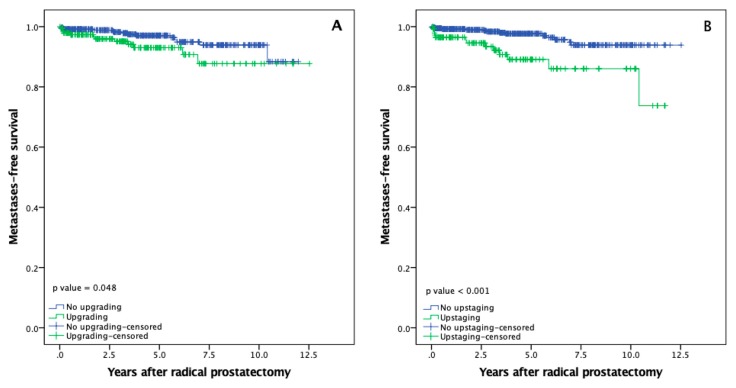
Metastases-free survival after radical prostatectomy: (**A**) Metastases-free survival according to upgrading; (**B**) Metastases-free survival according to upstaging.

Metastatic disease was diagnosed to 4.0% (27/676) of PCa patients. According to upgrading and upstagind, metastases were diagnosed to 6.1% (12/197) of PCa patients whose cancer upgraded post RP, while to 3.1% (15/479) of patients with no upgrading (*p* = 0.074). Mean metastasis-free survival was 11.5 (95% CI: 10.9–12.1) and 11.4 (95% CI: 11.1–11.7) years for patients with and without upgrading, respectively (Figure 2A, *p* = 0.048).

Upstaging after RP was also associated with metastastatic disease, where 8.7% (13/149) of PCa patients undergoing upstaging and 2.7% (14/527) of patients without upstaging developed metastases (*p* = 0.001). Mean mestastasis-free survival was 10.3 (95% CI: 9.6–11.1) years for patients with upstaging, as compared with 12.1 (95% CI: 11.8–12.3) years for patients with no upstaging (Figure 2B, *p* < 0.001)

In multivariate logistic regression analysis upstaging showed the highest OR for metastatic disease (3.40 (1.52-7.61), *p* = 0.003), followed by PSA (1.05 (1.01-1.08), *p* = 0.004), while upgrading was removed from the model.

### 3.5. Overall and Cancer Specific Survival

**Figure 3:**
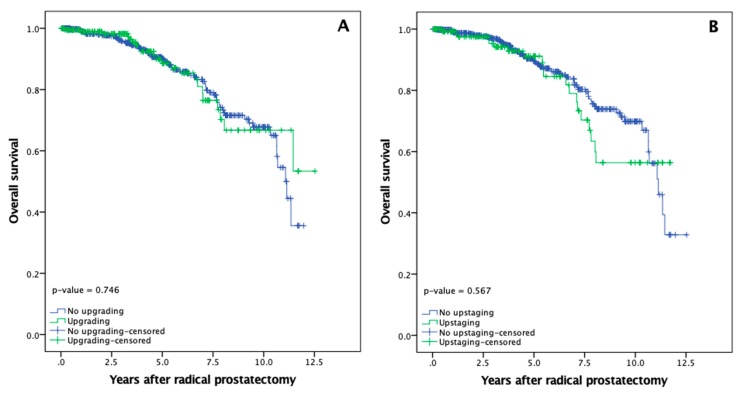
Prostate cancer overall survival rates after radical prostatectomy: (**A**) Overall survival according to upgrading; (**B**) Overall survival according to upstaging.

Mean OS for patients with and without upgrading was 10.2 (95% CI: 9.3–11.0) and 9.7 (95% CI: 9.3–10.2) years, while five and ten-year OS rates were comparable in both groups: 88.6%, 66.7% and 90.1%, 67.7%, respectively (Figure 3A, *p* = 0.746). Similar OS results were observed in upstaged and non-upstaged PCa cases, where mean overall survival was 9.3 (95% CI: 8.5–10.2) and 10.0 (95% CI: 9.5–10.5) years, while five and ten-year OS did not differ significantly: 91.2%, 56.4% and 89.5%, 69.9%, respectively (Figure 3B, *p* = 0.567).

**Figure 4:**
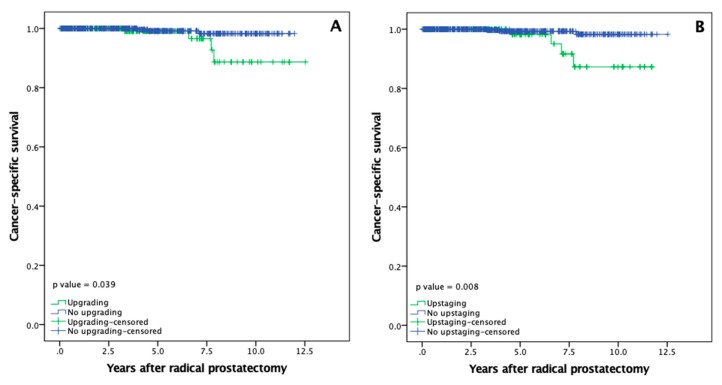
Prostate cancer-specific survival rates after radical prostatectomy: (**A**) Cancer-specific survival according to upgrading; (**B**) Cancer-specific survival according to upstaging.

For patients with and without PCa upgrading mean CSS was 11.9 (95% CI: 11.3–12.5) and 11.9 (95% CI: 11.7–12.0) years. Five-year CSS did not differ between both cohorts (99.1% vs. 99.1%), while ten-year CSS rate was significantly lower (88.7% vs. 98.3%) in patients who underwent pathological upgrading after RP (Figure 4A; *p* = 0.039). Mean CSS for upstaged and non-upstaged PCa was 11.1 (95% CI: 10.5–11.7) and 12.4 (95% CI: 12.3–12.5) years. No differences were also observed at the five-year mark (98.2% vs. 99.3%), while upstaging was associated with inferior ten-year CSS rates after RP (87.3% vs. 98.3%; Figure 4B; *p* = 0.008).

## 4. Discussion

PCa with high-levels of molecular and morphological diversity is an extremely heterogeneous neoplasm, ranging from clinically indolent to metastatic and life-threatening disease [2]. Therefore, accurate assessment of tumour characteristics at diagnosis is essential for optimal disease management. The D’Amico classification is the most commonly used criterion for the definition of PCa [13], however high rates of upgrading (24%–41%) and upstaging (29%–34%) have been reported after RP so far [6,14,15,16]. Discrepancies between prostate biopsy results and final pathological assessment of prostatectomy specimens may be attributed to diagnostic problems, especially when a higher Gleason grade tumour is missed on the needle biopsy or insufficient biopsy material is available for pathological examination [17]. In the present study, as in our previous research with a larger cohort [7], upgrading and upstaging have been observed in 29% and 22% of PCa patients, respectively.

The clinical and prognostic significance of PCa upgrading and upstaging remains controversial. According to our findings, patients undergoing upgrading or upstaging after RP are 1.5 times more likely to have a PSM on pathological specimen. It is generally known that PSM occur due to the biology of PCa and are associated with RP for high-risk disease as well as surgical experience [18]. Our results could be partly explained by these findings, while RP was performed by 8 different surgeons with different surgical and clinical experience. The highest PSM rates (30–45%) were detected for low-volume surgeons (<20 cases annually), while significantly lower PSM rates (10–26%) were detected for urologists with high surgical experience. Adverse cancer-specific features definitely increase the risk for PSM [19], especially in upgrading and upstaging settings when surgeons are facing the disease clinically suspected to have low-risk of progression [20].

It has been shown that downgrading is associated with better BCR-free survival [21], while upgrading increases the risk for BCR, which dramatically varies depending on PCa clinical characteristics [14]. According to our findings, both upgrading and upstaging significantly increase the risk for BCR (1.8 and 2.1 times, respectively), while patients with clinically diagnosed intermediate and high-risk disease carry the highest risk. Different risk for BCR could be explained by different upgrading categories, i.e., the vast majority of low-risk patients (cISUP 1) are upgraded to intermediate-risk disease (pISUP 2; in the present study 86.3% (145/168)), while intermediate-risk PCa cases (cISUP 2) are upgraded to an even higher-risk disease, i.e., pISUP 3 and higher.

The association between BCR and progression to metastatic disease and death of PCa is well documented in the literature [22], thus as the endpoints of our study MFS, OS and CSS were analysed. According to our findings, MFS was significantly shorter in patients with upgrading and upstaging, while grade and stage increase after RP did not reveal any impact on OS, but was associated with inferior ten-year CSS results: 89% vs. 98% for upgrading, and 87% vs. 98% for upstaging. Our findings are consistent with other investigators, where inferior CSS results have been reported for patients undergoing upstaging [23] and upgrading to more aggressive (pISUP ≥ 4) disease [24].

Disease upgrading and upstaging after radical treatment are raising the issue about serious diagnostic problems in PCa and are often the rationale for costly imaging or genomic studies, especially when active surveillance is considered. Several nomograms have been suggested to predict the probability of pathologic upgrading in patients with low-risk disease [25,26,27], however most of them are based on randomised biopsies and have limited value in counselling patients who are candidates for definitive therapy. Novel molecular biomarkers and genomic classifiers, containing molecular information from all tumour foci and reflecting PCa heterogeneity, have shown accuracy in predicting PCa aggressiveness and may provide valuable information for improved diagnostics [7,28,29,30].

Our research supports current concepts about limitations of preoperative PCa assessment and provides important evidence of some controversies regarding clinical implications of PCa upgrading and upstaging. However, we must acknowledge several limitations of the present study.

It has been published that prostate multiparametric MRI (mpMRI)-targeted biopsies are associated with less pathological upgrading at radical prostatectomy [31]. The biggest limitation of our study is that mpMRI was not routinely performed on our population. The study was initiated at 2008 when prostate MRI was rarely used for PCa diagnostics and the results of pre-operative MRI were available only for 48 of our patients, thus were not taken into consideration. Secondly, although the clinical data were maintained prospectively, the patients’ follow-up and survival were analysed in a retrospective way. Thirdly, this is a single-institution experience, therefore, external validation is mandatory.

## 5. Conclusions

Our findings supplement prevailing concepts that the currently used risk stratification models are associated with a substantial number of misdiagnosis in the light of PCa heterogeneity. Upgrading and upstaging after RP are associated with inferior surgical results, substantial higher risk of BCR and inferior rates of important oncological outcomes. This supports the idea that clinical risk is an important factor and all these findings should be considered when counselling PCa patients in order to focus efforts on improving oncologic surgical care with the goal to improve patient outcomes.

## Figures and Tables

**Table 1:** Clinico-pathological characteristics of prostate cancer patients.

Variable	PCa Patients (*N* = 676)
**Age at surgery, years**	
Mean (±SD)	62.1 (7.8)
**Preoperative PSA, ng/mL^a^**	
Mean (±SD)	8.2 (7.2)
**Prostate size, g^b^**	
Mean (±SD)	52.5 (23.3)
**cISUP grade group, *n* (%)**	
1	459 (67.9)
2	152 (22.5)
3	40 (5.9)
4	23 (3.4)
5	2 (0.3)
**pISUP grade group, *n* (%)**	
1	312 (46.1)
2	284 (42.0)
3	58 (8.6)
4	8 (1.2)
5	14 (2.1)
**cT stage, *n* (%)**	
≤cT1c	406 (60.1)
cT2a	7 (1.0)
cT2b	84 (12.4)
cT2c	120 (17.8)
cT3a	50 (7.4)
cT3b	9 (1.3)
cT4	0 (0.0)
**pT stage, *n* (%)**	
pT2a	41 (6.1)
pT2b	6 (0.9)
pT2c	448 (66.3)
pT3a	105 (15.5)
pT3b	74 (10.9)
pT4	2 (0.3)
**pN stage, *n* (%)**	
pN0	653 (96.6)
pN+	23 (3.4)
**Time from biopsy to RP, days^c^**	
Mean (±SD)	114.7 (186.5)

Abbreviations: cISUP = clinical ISUP grading; cT = clinical T-staging; ISUP = International Society for Urological Pathology; N = regional lymph node staging according to TNM classification; pISUP = pathological ISUP grading; RP = radical prostatectomy; pN = pathological N-staging; pT = pathological T-staging; PCa = prostate cancer; PSA = prostate-specific antigen; SD = standard deviation; T = local tumour staging according to TNM classification. ^a^ PSA missing in 4 patients. ^b^ Prostate size missing in 6 patients. ^c^ Time from biopsy to RP missing in 51 patients.

**Table 2:** Univariate and multivariate logistic regression analysis of the associations between clinico-pathological characteristics and biochemical recurrence.

Variable	Univariate			Multivariate		
	Odds ratio	95% CI	*P*-Value	Odds ratio	95% CI	*P*-Value
PSA, ng/mL	1.10	(1.07–1.13)	**<0.001**	1.09	(1.05–1.13)	**<0.001**
Prostate size, g	0.99	(0.99–1.00)	0.182	0.99	(0.98–1.00)	0.057
PSM	3.27	(2.28–4.69)	**<0.001**	2.29	(1.55–3.40)	**<0.001**
Upgrading*	2.28	(1.59–3.28)	**<0.001**	1.92	(1.29–2.86)	**0.001**
Upstaging	2.97	(2.02–4.37)	**<0.001**	2.14	(1.39–3.27)	**<0.001**

Abbreviations: CI = confidence interval; cISUP = clinical International Society of Urological Pathology (ISUP) group; PSA = prostate-specific antigen; PSM = positive surgical margin. *All cISUP grade groups were included. Statistically significant *p*-values (*p* <0.050) are marked in bold.
